# Nanovesicles Loaded with *Origanum onites* and *Satureja thymbra* Essential Oils and Their Activity against Food-Borne Pathogens and Spoilage Microorganisms

**DOI:** 10.3390/molecules26082124

**Published:** 2021-04-07

**Authors:** Giulia Vanti, Ekaterina-Michaela Tomou, Dejan Stojković, Ana Ćirić, Anna Rita Bilia, Helen Skaltsa

**Affiliations:** 1 Department of Chemistry “Ugo Schiff”, University of Florence, Via Ugo Schiff 6, 50019 Sesto Fiorentino, FI, Italy; giulia.vanti@unifi.it (G.V.); ar.bilia@unifi.it (A.R.B.); 2 Faculty of Pharmacy, Department of Pharmacognosy & Chemistry of Natural Products, National & Kapodistrian University of Athens, Panepistimiopolis, Zografou, 157 71 Athens, Greece; ktomou@pharm.uoa.gr; 3 Department of Plant Physiology, Institute for Biological Research “Siniša Stanković”—National Institute of Republic of Serbia, University of Belgrade, Bulevar Despota Stefana 142, 11000 Belgrade, Serbia; dejanbio@yahoo.com (D.S.); anciranci@yahoo.co.uk (A.Ć.)

**Keywords:** *Origanum onites* essential oil, *Satureja thymbra* essential oil, GC-MS analysis, propylene glycol-nanovesicles, TEM, antimicrobial efficacy, HaCaT cytotoxicity, Symi island

## Abstract

Food poisoning is a common cause of illness and death in developing countries. Essential oils (EOs) could be effective and safe natural preservatives to prevent and control bacterial contamination of foods. However, their high sensitivity and strong flavor limit their application and biological effectiveness. The aim of this study was firstly the chemical analysis and the antimicrobial evaluation of the EOs of *Origanum onites* L. and *Satureja thymbra* L. obtained from Symi island (Greece), and, secondly, the formulation of propylene glycol-nanovesicles loaded with these EOs to improve their antimicrobial properties. The EOs were analyzed by GC-MS and their chemical contents are presented herein. Different nanovesicles were formulated with small average sizes, high homogeneity, and optimal ζ-potential. Microscopic observation confirmed their small and spherical shape. Antibacterial and antifungal activities of the formulated EOs were evaluated against food-borne pathogens and spoilage microorganisms compared to pure EOs. Propylene glycol-nanovesicles loaded with *O. onites* EO were found to be the most active formulation against all tested strains. Additionally, in vitro studies on the HaCaT cell line showed that nanovesicles encapsulated with EOs had no toxic effect. The present study revealed that both EOs can be used as alternative sanitizers and preservatives in the food industry, and that their formulation in nanovesicles can provide a suitable approach as food-grade delivery system.

## 1. Introduction

Lamiaceae is a well-known family of aromatic herbs, diffused in many regions of the world. Numerous plants that belong to this family are used for medicinal purposes and in perfumery, and they are extensively applied to impart flavor and aroma to foods. Most of them are rich in essential oils (EOs) [1,2] which consist of complex mixtures of volatile, liquid, odorous, flavorsome and strongly active compounds. Due to their various biological properties, principally antioxidant and antimicrobial, they have been widely used since the Middle Ages and currently, they may have several applications in different fields, from medicine and cosmetics to food. However, their high volatility and low stability to direct exposure to light, oxygen, heat, and humidity can limit their potential.

Nanocarriers represent an innovative challenge to optimize the essential oil formulation, overcoming the main limitations [3]. Liposomes are vesicles containing an aqueous phase entirely surrounded by a bilayer constituted of phospholipids and cholesterol, which can definitely load the essential oils. The resulting nanocarrier is stable and efficient, easily and safely produced, capable of stabilizing the essential oil, modulating its release and optimizing its activity [4]. Recently, a commercial *Melissa officinalis* L. essential oil was successfully formulated in glycerosomes, being very active in vitro in inhibiting HSV type 1 infection of mammalian cells, without producing cytotoxic effects [5]. In addition, Risaliti and coworkers (2019) demonstrated enhanced antioxidant, anti-inflammatory and antimicrobial activities of the essential oils of *Rosmarinus officinalis* L. and *Salvia triloba* L. plants, when formulated in liposomes [6]. The same authors further improved antifungal properties against 10 different drug-resistant *Candida* strains of *Artemia annua* L. EO loaded in nanoliposomes [7].

EOs obtained from aromatic and medicinal plants, as well as their components have largely demonstrated antibacterial and antifungal properties against a wide range of microbial pathogens, including numerous food-borne pathogens and key spoilage bacteria [8,9]. Food poisoning is considered as one of the most common causes of illness and death in developing countries, which are associated with bacterial contamination especially from Gram-negative bacteria, mainly represented by *Salmonella typhi*, *Escherichia coli* and *Pseudomonas aeruginosa* [10]. Among Gram-positive bacteria, *Staphylococcus aureus* and *Bacillus cereus* are the most causative agents of food-borne illnesses or food spoilage [11]. Over recent years, synthetic preservatives are largely used to successfully prevent and control bacterial contamination of food, but their use can result in unwanted chemical residues in food and feed chains. In addition, there is an increasing resistance of these pathogens to the synthetic preservatives [12]. Hence, the discovery of alternative preservatives, mainly originated by natural sources have been increased during recent years. EOs are reported as potentially effective and safe natural food preservatives by many publications [8]. Although EOs possess good antimicrobial properties, they have potential limitations for commercial applications due to their physical and organoleptic properties such as strong flavor, volatility and chemical instability. Consequently, different types of food-grade delivery systems, mainly nano-emulsions [13], but also innovative nanocochleates [14] have now been evaluated for improving their performance as antimicrobials.

Plants of genus *Origanum* L. and *Satureja* L. are well-known aromatic herbs, widely used in traditional and modern medicine, as well as in food and cosmetics. Many of them are referred with the common name “oregano” [15]. Among the oregano plants, *O. vulgare* L. is one of the most popular aromatic species, including six subspecies; *O. vulgare* subsp. *glandulosum* (Desf.) Ietsw., *O. vulgare* subsp. *gracile* (K. Koch) Ietsw., *O. vulgare* subsp. *hirtum* (Link) Ietsw., *O. vulgare* subsp. *virens* (Hoffmanns. and Link) Ietsw., *O. vulgare* subsp. *viridulum* (Martrin-Donos) Nyman, and *O. vulgare* subsp. *vulgare* [16]. Strikingly, the two most commercially important oregano herbs are considered the *O. vulgare* subsp. *hirtum* (known as “Greek oregano”) and *O. onites* L. (known as “Turkish oregano”, “Island oregano” or “Cretan oregano”) [17,18]. The latter herb is a narrowly distributed East Mediterranean species, occurring mainly in Turkey and Greece. The interest for this species is due to the high essential oil yield and carvacrol content (69.0–92.6%) [18,19]. *S thymbra* L. is distributed in the Mediterranean region and its smell is close to that of oregano [20]. Its essential oil is rich in monoterpene derivatives such as γ-terpinene, *p*-cymene and carvacrol [21]. Both EOs exert a broad range of pharmacological properties, especially antimicrobial activity, which are attributed to their chemical constituents (i.e., carvacrol, γ-terpinene and *p*-cymene) [22,23].

In this study, *O. onites* and *S. thymbra* EOs were isolated and analyzed by GC-MS, and for the first time, they were formulated in propylene glycol-nanovesicles, proposed as safe and food-grade delivery systems. Different nanovesicles were developed and optimized in terms of size and essential oil loading and were fully characterized as regards physical and chemical parameters. Successively, they were investigated for the antimicrobial activity against a panel of different bacteria and fungi, comparing the activity with that of pure essential oils. The safety profile was assessed by in vitro test on HaCaT cell line.

## 2. Results and Discussion

### 2.1. Chemical Composition of OOEO and STEO

The yield (*v*/*w*) of the EO obtained from *O. onites* (OOEO) was 3.0%. Overall, thirty-five compounds, representing 100.0% of the OOEO, were identified. In particular, the main components were carvacrol (66.0%), *p*-cymene (7.9%), γ-terpinene (4.9%) and borneol (2.8%). Furthermore, reasonable levels of β-bisabolene (2.3%), myrcene (2.1%), α-terpinene (2.0%) and terpinen-4-ol (1.7%) were also detected, while α-pinene and thymol were found in low percentages (both 1.0%) (Table 1). Oxygenated monoterpenes (74.1%) comprised the major chemical group of the OOEO, followed by monoterpene hydrocarbons (21.2%) and sesquiterpene hydrocarbons (4.1%), (Table 1). It is well-known that the OOEO is high rich in carvacrol content (up to 90%) [18,19,23,24,25]. Vokou and coworkers (1988) investigated the EOs of *O. onites*, originated from different parts of Greece, underlying the high yields of EOs from south-eastern islands (Halki, Symi and Tilos) [26]. Given that different OOEO chemotypes were reported based on their main volatile constituents [23], our studied OOEO revealed a carvacrol chemotype. We should point out that linalool was determined in a low amount (0.4%). Previous studies discussed the distinction among the *O. vulgare* ssp. *hirtum* and *O. onites*, mentioning that the EO of the latter species is poor in thymol and/or *p*-cymene, whereas its borneol content ranges more than 2.0% [18,27]. Our findings comply with this distinction since thymol concentration was low (1.0%) and borneol was found in higher level (2.8%).

In the EO of *S. thymbra* (STEO; yield 2.8% *v*/*w*) were identified twenty-nine chemical constituents, representing 99.9% of the total amount (Table 2). These constituents were grouped into oxygenated monoterpenes (50.0%), monoterpene hydrocarbons (41.9%), sesquiterpene hydrocarbons (7.7%), oxygenated sesquiterpenes (0.2%) and aliphatic alcohols (0.1%) (Table 2). Precisely, the main compounds were carvacrol (46.0%), γ-terpinene (19.7%), *p*-cymene (7.6%), β-caryophyllene (7.0%) and α-terpinene (5.1%). Other constituents in lower concentrations were myrcene (2.5%), α-thujene (2.4%), α-pinene (1.6%) and linalool (1.3%). Our results are in accordance with previous studies in EO of *S. thymbra* [20,22,25,28].

Comparing the two investigated EOs, carvacrol was the dominant component, while thymol was detected in very low concentrations (<1.0%) in both samples. We also observed that the high carvacrol (66.0%) content was related to low amount of γ-terpinene (4.9%) in the EO of *O. onites*, whereas the EO of *S. thymbra* demonstrated a relatively lower concentration of carvacrol (46.0%) followed by a high content of γ-terpinene (19.7%). This observation is congruous with a previous study [25] and could be attributed to biosynthetic pathways, considering that γ-terpinene and *p*-cymene are biosynthetic precursors of carvacrol. In addition, the two samples presented similar chemical groups. Both plants were rich in EOs which is directly related to the distinct environmental and geographical conditions of Symi island which belongs to the Dodecanese island complex (SE Aegean region).

### 2.2. Development and Optimisation of Nanovesicles Loaded with OOEO and STEO

EOs of *O. onites* (OOEO) and *S. thymbra* (STEO) were formulated using diverse nanovesicles. Firstly, OOEO was encapsulated in conventional liposomes using PBS as dispersant medium and phosphatidylcholine (P90G) plus cholesterol in different ratios, trying to optimize average diameter and polydispersity of the vesicles. An amount of 10 mg/mL of OOEO was found to be the optimal concentration to obtain stable formulations without OOEO extrusion from the system, but vesicles resulted not homogeneous. Accordingly, other types of vesicles were approached, in particular those obtained by mixing membrane components with a water-soluble, non-volatile organic solvent, such as a polyol. The resulting nanovesicles are physiologically suitable even when administered intravenously into the human body. In particular, both glycerol and propylene glycol are among the most widely used raw materials in food, cosmetic and pharmaceutical industries. Glycerol shows excellent solubility in water, and, due to its GRAS status, it is very safe to use [29]. Propylene glycol is a colorless, odorless and completely water-soluble solvent very similar to glycerol, also having the GRAS status and high safety [30]. Glycerosomes were prepared according to the recent publication by Vanti and coworkers [5]. Different experimental conditions were tested, as reported in the experimental section. Finally, the lipid film was constituted of P90G (600 mg) plus cholesterol (10 mg). It was hydrated using a 5% *v*/*v* glycerol/water solution, and OOEO (10 mg/mL) or STEO (10 mg/mL) or OOEO plus STEO (5 mg/mL plus 5 mg/mL), were added in this step, obtaining *O. onites* essential oil-loaded glycerosomes (OO-GS), *S. thymbra* essential oil-loaded glycerosomes (ST-GS) and *O. onites* plus *S. thymbra* essential oil-loaded glycerosomes (OOST-GS). In parallel, different nanovesicles were prepared hydrating the lipid film with a 1% *v*/*v* propylene glycol/water solution and obtaining propylene glycol-nanovesicles (PGV) loaded with: *O. onites* essential oil (OO-PGV), *S. thymbra* essential oil (ST-PGV) and *O. onites* plus *S. thymbra* essential oil (OOST-PGV). All the samples were analyzed by light scattering techniques and they showed small dimensions, low polydispersity index (PdI) and good ζ-potential (Table 3 and Table 4). The higher standard deviation related to the average sizes of OO-GS and OO-PGV indicates a lower repeatability of sample preparation. However, the application of nanovesicles in food products is not limited by their average dimensions and all the developed formulations have suitable physical characteristics as delivery systems of food preservatives.

**Figure 1:**
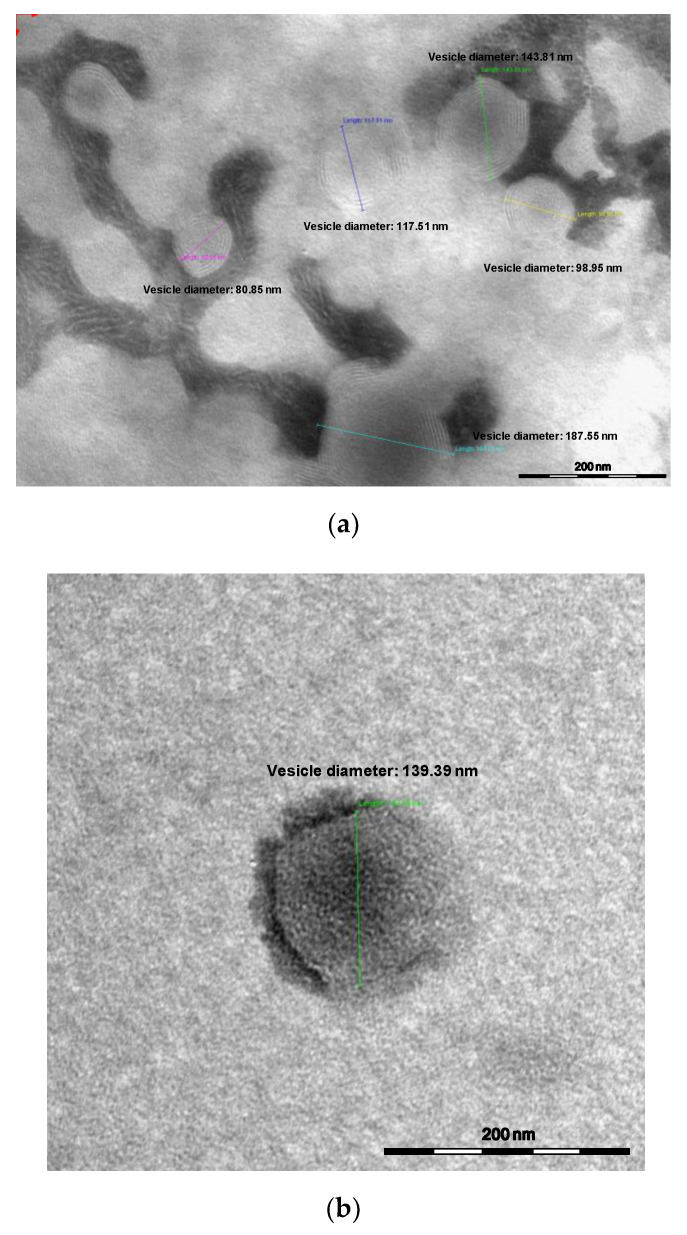
Pictures of (**a**) glycerosomes loaded with *O. onites* plus *S. thymbra* essential oils (OOST-GS) and (**b**) propylene glycol-nanovesicles loaded with *O. onites* plus *S. thymbra* essential oils (OOST-PGV), obtained by transmission electron microscope (TEM) analysis.

The combination of mechanic stirrer and ultrasonic bath during the two hydration processes allowed to obtain homogenous and stable formulations, without any further optimization step. Glycerosomes and PG-nanovesicles loaded with OOEO plus STEO were analyzed by transmission electron microscope (TEM, Figure 1a,b). The obtained micrographs showed vesicles with spherical shape and several lamellae, mainly visible in glycerosomes (Figure 1a), and with dimensions in accordance with those obtained by DLS.

In addition, the encapsulation efficiency (EE) of OOEO and STEO, expressed as percentage of carvacrol, the marker constituent, was evaluated by HPLC-DAD and resulted high in both glycerosomes (between ca. 73% and ca. 77%) and PG-nanovesicles (between ca. 77% and 83%).

### 2.3. Antibacterial and Antifungal Activities

Results of the evaluation of antibacterial and antifungal activity of pure and formulated OOEO/STEO are reported in Table 5 and Table 6. All tested samples possessed significant antimicrobial effects. The best antimicrobial activities of formulated OOEO/STEO against all tested bacteria and fungi were observed for OO-PGV. The antibacterial minimum inhibitory concentration (MIC) of OO-PGV ranged from 1.00 to 4.00 mg vesicles/mL, while the minimum bactericidal concentrations (MBCs) were within the range of 2.00 to 8.00 mg vesicles/mL. MIC and MBC of essential oils formulated in nanovesicles, reported in Table 5, were calculated taking into account that the amounts of EOs loaded in the nanovesicles were 10 mg EO/g of vesicles. The pure OOEO exhibited stronger antibacterial potential with MICs at 0.0002–0.002 mg/mL and MBCs at 0.0003–0.0025 mg/mL compared to standard antibiotic Streptomycin and formulated OOEO used as reference compounds. The most sensitive bacterial species was *S. aureus*, while *E. coli* was the resistant one among all the tested bacteria. MIC and MFC were reported in Table 6: for OOEO, MICs were in the range 0.0002–0.001 mg/mL against all tested fungi, and MFCs varied within the range of 0.0003–0.0012 mg/mL. Antifungal potential of OOEO was higher than that of formulated OOEO and reference drug Ketoconazole. The most sensitive fungus appeared to be *P. verrucosum*, while both *Candida* species were found to be resistant. In conclusion, the activity of both pure OOEO and STEO was more prominent when compared to the activity of corresponding amounts of formulated EOs. However, this lower antibacterial and antifungal effectiveness of formulated EOs with respect to pure EOs is mainly due to the prolonged release properties of the EOs loaded in the nanovesicles, as described by other studies on in vitro activity of essential oils formulated in glycerosomes and liposomes [5,31].

### 2.4. Cytotoxicity on HaCaT Cell Line

The cytotoxic effect of pure and formulated OOEO/STEO (Table 7) was assessed on the HaCaT cell line, a spontaneously transformed aneuploid immortal keratinocyte cell line from adult human skin, a very sensible cell line used as an effective in vitro alternative for an initial orientating screening of safety issues of substances. Blank-GS and Blank-PGV samples showed no toxicity towards HaCaT up to 500 µg/mL, which is indicative of carrier low cytotoxicity. All the investigated samples showed to be weakly cytotoxic towards this cell line. The results showed that OO-GS sample showed the highest cytotoxic effect on the human immortalized keratinocytes, followed by OOST-GS (combination of essential oils loaded in glycerosomes). OO-PGV (propylene glycol-nanovesicles loaded with *O. onites* essential oil) was the only sample with a cytotoxic effect below 500 µg/mL. Although weak cytotoxicity could be acknowledged for some samples, further application of propylene glycol-nanovesicles should be considered when formulating antimicrobial preparations, since this carrier expressed no toxicity to immortalized cell line. These safety data are encouraging for further safety studies to demonstrate the safe use of the developed nanovesicles.

## 3. Materials and Methods

### 3.1. Plant Materials

The fresh aerial parts of the wild plants *Origanum onites* L. and *Satureja thymbra* L. were collected from Symi island (SE Aegean, Greece) in summer 2018. The plant materials were authenticated by Associate Prof. Th. Constantinidis; Voucher specimens were deposited to a personal Herbarium of the Department of Pharmacognosy and Chemistry of Natural Products, School of Pharmacy, NKUA (Voucher Specimen Numbers: Tomou and Skaltsa 004/005).

### 3.2. Chemicals

Phosphatidylcholine (Phospholipon 90G, P90G) was purchased from Lipoid AG (Cologne, Germany) with the support of the Italian agency AVG srl. Cholesterol 95%, dichloromethane, methanol and acetonitrile were purchased from Sigma-Aldrich (Milan, Italy); vegetable glycerol Eur Ph. and propylene glycol Eur Ph. were purchased from Galeno srl (Prato, Italy). Ultrapure water was produced by a synergy UV Simplicity water purification system provided by Merck KGaA (Molsheim, France). Phosphotungstic acid (PTA) was purchased from Electron Microscopy Sciences (Hatfield, PA, USA).

### 3.3. Hydrodistillation and Identification of OOEO and STEO by Gas Chromatography–Mass Spectrometry (GC-MS)

Air-dried parts (40.0 g) of each plant were cut into small pieces and subjected separately to hydrodistillation for 2 h, using a modified Clevenger type apparatus with a water-cooled oil receiver to reduce artifacts produced during distillation by over-heating according to Hellenic Pharmacopoeia [32]. The EOs were obtained by gas chromatography (GC) grade *n*-pentane and dried over anhydrous sodium sulfate and stored at −20 °C. The compositions of the volatile constituents were established by GC/MS analyses, performed on a Hewlett-Packard 7820A-5977B MSD system operating in EI mode (70 eV) equipped with a split/splitless injector, using a fused silica HP-5 MS capillary column (30 m × 0.25 mm I.D., film thickness: 0.25 μm). Helium was used as a carrier gas at a flow rate of 2.0 mL/min. The oven temperature was increased from 60 to 300 °C at a rate of 3 °C/min, and subsequently held at 300 °C for 10 min. Injection was at 220 °C in a split ratio 1:5. Injection volumes of each sample were 2 μL. Retention indices for all compounds were determined according to the Van den Dool approach [33], using *n*-alkanes as standards. The identification of the components was based on comparison of their mass spectra with those of Wiley and NBS/NIST Libraries and those described by Adams (2017) [34], as well as by comparison of their retention indices with literature data. In many cases, the essential oils were subjected to co-chromatography with authentic compounds (Fluka, Sigma). Semi-quantification through peak area integration from GC peaks was applied to obtain the component percentages. The analyses were carried out twice for each sample.

### 3.4. HPLC-DAD Analysis

Quantitative analyses of OOEO and STEO in nanovesicles was based on the determination of carvacrol, the most abundant and marker constituent of both essential oils. Analyses were carried out using a 1100 High Performance Liquid Chromatograph (HPLC) equipped with a diode array detector (DAD), by Agilent Technologies Italia Spa (Rome, Italy). The chromatographic analyses were performed using a reverse-phase column Luna C-18 100 Å (250 × 4.6) mm, 5 µm particle size, maintained at 25 °C; and the chromatograms were acquired at 276 nm [35]. A gradient elution method, with 1 mL/min flow rate, was applied, using (A) acetonitrile and (B) formic acid/water (pH 3.2) as mobile phases. The analytical method was: 0–3 min 30–30% (B), 3–10 min 30–80% (B), 10–15 min 80–80% (B), 15–20 min 80–95% (B), 20–22 min 95–95% (B), 22–27 min 95–30%. The calibration curve was prepared using a 0.001 μL/μL standard solution of carvacrol in methanol and successive dilutions. The coefficient of determination (R^2^) of carvacrol calibration curve was 0.9999.

### 3.5. Preparation of Vesicles Loaded with OOEO and STEO

*O. onites* and *S. thymbra* essential oils (OOEO and STEO) were formulated in lipid nanovesicles by the lipid film hydration method [36], in two steps [5,37]. Different amounts of phosphatidylcholine (P90G) and cholesterol were tested for the development of the formulation, and the experimental conditions of the preparation were optimized varying media and time of hydration, as well as the use of ultrasonic bath and/or mechanic stirrer, as reported in Table 8. The selected formulations were prepared with 600 mg of phosphatidylcholine and 10 mg of cholesterol dissolved in dichloromethane, using the ultrasonic bath for 1 min in order to improve the dissolution. Subsequently, evaporation of dichloromethane was carried out using the rotavapor for 20 min at 30 °C, in order to obtain a homogenous lipid film on the internal surface of the flask. At this stage, OOEO (100 μL) or STEO (100 μL) or OOEO plus STEO (50 μL + 50 μL) were added to the flask and the lipid film was hydrated with 5 mL of 5% *v*/*v* glycerol/water solution (glycerosomes, GS) or 1% *v*/*v* propylene glycol/water solution (propylene glycol-nanovesicles, PG-nanovesicles, PGV), by using the mechanic stirrer for 30 min at 25 °C and immersing the flask in the ultrasonic bath, as shown in Table 8, Table 9 and Table 10. Then, a further 5 mL of the selected dispersant medium was added, and the dispersion was mechanically shaken for additional 30 min, at 25 °C, in the ultrasonic bath.

### 3.6. Physical Characterization of Nanovesicles Loaded with OOEO and STEO

Average hydrodynamic diameter (nm), polydispersity index (PdI) and ζ-potential (mV) of the developed nanovesicles were measured by Dynamic and Electrophoretic Light Scattering, DLS/ELS (Zetasizer Nanoseries ZS90) by Malvern instrument (Worcestershire, UK), at 25 °C, with a scattering angle of 90 °C [38,39]. Glycerosomes and PG-nanovesicles loaded with the essential oils were diluted with ultrapure water before measurements, in order to achieve a suitable scattering intensity. Successively, the two systems loaded with OOEO plus STEO were observed by transmission electron microscope, TEM (CM12 TEM, PHILIPS, Eindhoven, The Netherlands) equipped with an OLYMPUS Megaview G2 camera, with an accelerating voltage of 80 kV. A drop of sample, 5-folds diluted in water, was applied and dried by desiccation on a carbon film copper grid and it was counterstained with 1% *w*/*v* of phosphotungstic acid solution for 3 min [40]. Then, the sample was examined at different amplifications.

### 3.7. Chemical Characterization of Nanovesicles Loaded with OOEO and STEO

Encapsulation efficiency (EE) and total recovery (R) of OOEO and STEO loaded inside nanovesicles were evaluated in terms of carvacrol content, the marker constituent of both the essential oils. *EE* was calculated according to the following equation:

(1) $$EE=\left(\frac{encapsulated\;carvacrol}{initial\;carvacrol}\right)\times 100$$

where *encapsulated carvacrol* is the concentration of the single component after the purification step. In fact, vesicles were purified from free EOs by the dialysis bag method [41], using Spectra/Por^®^ regenerated cellulose membranes with 3.5 KDa molecular weight cut-off, by Repligen Europe B.V. (Breda, The Netherlands). The dialysis bag was stirred in 1 L of ultrapure water, at room temperature for 1 h. After that, the purified formulations were diluted in methanol, in order to break vesicles and release the encapsulated EOs. Samples were centrifuged at 14,000 rpm for 10 min and supernatants were analyzed by HPLC-DAD. OOEO and STEO total recovery (*R*) was determined using the same procedure without the purification step by dialysis, and it was calculated according to the following equation:

(2) $$R=\left(\frac{total\;recovered\;carvacrol}{initial\;carvacrol}\right)\times 100$$

where *total recovered carvacrol* is the concentration of the single component after the preparation of the formulation, determined by chromatographic analysis.

### 3.8. Determination of Antibacterial and Antifungal Activity

The Gram-positive bacteria *Bacillus cereus* (food isolate), *Staphylococcus aureus* ATCC 11632 and *Listeria monocytogenes* NCTC 7973, and the Gram-negative bacteria *Escherichia coli* ATCC 35210, *Pseudomonas aeruginosa* ATCC 27853 and *Salmonella enterica* subsp. *enterica* serovar Typhimurium ATCC 13311 were used in order to determine the potential antibacterial activity. For determination antifungal activity 6 strains of fungi were used: *Aspergillus fumigatus* ATCC 1022, *Aspergillus niger* ATCC 6275, *Trichoderma viride* IAM 5061, *Penicillium verrucosum* var. *cyclopium* (food isolate), *Candida albicans* (oral isolate) and *Candida krusei* (oral isolate) were tested for their susceptibility. The bacterial strains were cultured on solid Tryptic Soy agar (TSA), while micromycetes were cultured on solid malt agar (MA) and yeast were sustained on Sabouraud dextrose agar (SDA) medium. The cultures were sub-cultured once a month and stored at 4 °C for further utilization. All the tested microorganisms are deposited at the Mycological Laboratory, Department of Plant Physiology, Institute for Biological Research “Siniša Stankovic”—National Institute of Republic of Serbia, University of Belgrade, Serbia. The antimicrobial activity of samples was determined by the modified microdilution method [42,43]. The results were presented as minimum inhibitory concentrations (MICs) and minimum bactericidal/fungicidal concentrations (MBCs/MFCs). Streptomycin (Sigma-Aldrich S6501, St. Louis, MO, USA) and ketoconazole (Zorkapharma, Šabac, Serbia) were used as positive controls, and blank-glycerosome (Blank-GS) and blank-propylene glycol-nanovesicles (Blank-PGV) were used as negative control. All the experiments on antimicrobial activity were repeated in triplicate.

### 3.9. Evaluation of Cytotoxicity in HaCaT Cell Line

Crystal violet assay was used for determination of the antiproliferative effect, according to the previous protocol [44] with modifications. Antiproliferative effect of pure and formulated OOEO/STEO was analyzed on spontaneously immortalized human skin keratinocytes (HaCaT) cell line. Cell line was grown in high-glucose Dulbecco’s Modified Eagle Medium (DMEM) supplemented with 10% fetal bovine serum (FBS), 2 mM L-glutamine and 1% penicillin and streptomycin (Invitrogen) at 37 °C in 5% CO_2_. Twenty-four hours before treatment with the extract 1 × 10^4^ cells/well were seeded in a 96-well plate. After, the medium was removed, fresh medium supplemented with different concentrations of the extract and compound (6.25–400 μg/mL) dissolved in phosphate buffered saline was added to the cells. Control cells were grown in medium. Potassium dichromate (K_2_Cr_2_O_7_) was used as a positive control and PBS as negative control. The experiment was performed in triplicate for each condition and cells were incubated with the extract for 24 h. After that period, the medium was removed and the cells were washed twice with phosphate buffered saline (PBS), stained with 0.5% crystal violet staining solution and incubated for 15 min at room temperature. Afterwards, crystal violet was removed, the cells were washed in a stream of tap water and left to air-dry at room temperature for 24 h. The absorbance of dye dissolved in methanol was measured in a microplate reader at 590 nm (OD590). The results were expressed as IC_50_ (%) value in μg/mL. The criterion used to categorize the cytotoxic activity of pure and formulated OOEO/STEO to cancer cell lines was as follows: IC_50_ ≤ 20 µg/mL = highly cytotoxic, IC_50_ ranged between 21 and 250 µg/mL = moderately cytotoxic, IC_50_ ranged between 201 and 500 µg/mL = weakly cytotoxic, and IC_50_ > 501 µg/mL = no cytotoxicity. All analyses were performed in triplicate; each replicate was quantified also three times. Data were expressed as mean standard deviation, where applicable. In the cases where statistical significance differences were identified, the dependent variables were compared using Tukey’s honestly significant difference (HSD) test.

## 4. Conclusions

During recent years, synthetic preservatives are generally used to protect food against microorganisms. However, there is an urgent need to search new antimicrobials because of the increasing resistance against these microorganisms. EOs represent a valid alternative to synthetic preservatives in the food industry, and *Origanum* essential oil has been largely investigated as antimicrobial and antioxidant additive in food products [45]. However, in many cases their organoleptic impact in foodstuffs limits their usage. Techniques such as nanoencapsulation can address this problem. Thus, this study was designed in order to develop propylene glycol-nanovesicles loaded with EOs from *O. onites* and *S. thymbra* for the first time and to evaluate them as safe and food-grade delivery systems.

In this study, the chemical profiles of the EOs of *O. onites* and *S. thymbra* were identified. Oxygenated monoterpenes comprised the major chemical class in both EOs. In particular, the main components of *O. onites* EO were carvacrol (66.0%), *p*-cymene (7.9%), γ-terpinene (4.9%) and borneol (2.8%). Whereas, the principal compounds of *S. thymbra* EO were carvacrol (46.0%), γ-terpinene (19.7%), *p*-cymene (7.6%), β-caryophyllene (7.0%) and α-terpinene (5.1%). Afterwards, we succeeded to encapsulate the EOs in nanovesicles, which presented high homogeneity and optimal encapsulation efficiency either for *O. onites* or *S. thymbra*. Both pure EOs and formulated EOs were evaluated against different food-borne pathogens. The high antimicrobial activity of both EOs could be attributed to their chemical constituents, not only to the high concentration of carvacrol but also to the potential synergy of all the compounds. Our results showed that pure EOs were more active compared to the corresponding amounts of formulated EOs. We should point out that the lower antibacterial and anti-fungal effectiveness of formulated EOs with respect to pure EOs is assigned mainly to the prolonged release properties of the EOs loaded in the nanovesicles. Cytotoxicity was also tested in HaCaT cells.

In conclusion, the present study unveiled that the tested nanovesicles could represent potential biocontrol agents against fungal and bacterial food pathogens with promising GRAS status in mammalian systems, besides being an innovative and completely biodegradable approach for the prolonged and sustained release of the EO, preserving functional properties.

## Figures and Tables

**Table 1:** Chemical composition (% *v*/*v*) of *O. onites* essential oil.

No	Compounds	RI ^a^	Composition (%)
1	α-thujene	920	0.8
2	α-pinene	928	1.0
3	camphene	941	0.6
4	1-octen-3-ol	970	0.4
5	myrcene	985	2.1
6	α-phellandrene	1000	0.4
7	δ-3-carene	1004	0.2
8	α-terpinene	1010	2.0
9	p-cymene	1017	7.9
10	β-phellandrene	1021	0.8
11	(*E*)-β-ocimene	1040	0.1
12	γ-terpinene	1051	4.9
13	trans-sabinene hydrate	1072	0.3
14	α-terpinolene	1083	0.4
15	linalool	1091	0.4
16	α-campholenal	1120	0.1
17	trans-pinocarveol	1132	0.1
18	borneol	1163	2.8
19	terpinen-4-ol	1171	1.7
20	α-terpineol	1183	0.6
21	carvone	1235	0.1
22	carvacrol methyl ether	1241	0.4
23	linalyl acetate	1250	0.2
24	carvenone	1253	0.2
25	thymol	1285	1.0
26	carvacrol	1300	66.0
27	carvacrol acetate	1366	0.2
28	β-cubebene	1383	0.7
29	β-caryophyllene	1414	0.7
30	aromadendrene	1435	0.2
31	ledene	1495	0.1
32	β-bisabolene	1501	2.3
33	δ-cadinene	1518	0.1
34	spathulenol	1575	0.1
35	caryophyllene oxide	1580	0.1
	Total identification		100.0
**Grouped components (% *v*/*v*) of *O. onites* essential oil**			
**Classes of EO Constituents**		**%**	
Monoterpene hydrocarbons		21.2	
Oxygenated monoterpenes		74.1	
Sesquiterpene hydrocarbons		4.1	
Oxygenated sesquiterpenes		0.2	
Aliphatic alcohols		0.4	

^a^ RI: Retention Index, calculated against C_9_–C_24_ *n*-alkanes on the HP 5MS column capillary column.

**Table 2:** Chemical composition (% *v*/*v*) of *S. thymbra* essential oil.

No	Compounds	RI ^a^	Composition (%)
1	α-thujene	920	2.4
2	α-pinene	928	1.6
3	camphene	941	0.3
4	β-pinene	970	0.6
5	myrcene	985	2.5
6	3-octanol	987	0.1
7	α-phellandrene	1000	0.6
8	δ-3-carene	1004	0.2
9	α-terpinene	1010	5.1
10	p-cymene	1017	7.6
11	β-phellandrene	1021	0.9
12	(*E*)-β-ocimene	1040	0.1
13	γ-terpinene	1051	19.7
14	trans-sabinene hydrate	1072	0.3
15	α-terpinolene	1083	0.3
16	linalool	1091	1.3
17	borneol	1163	0.3
18	terpinen-4-ol	1171	0.9
19	thymol methyl ether	1232	0.7
20	carvone	1235	0.1
21	thymol	1285	0.3
22	carvacrol	1300	46.0
23	carvacrol acetate	1366	0.1
24	β-caryophyllene	1414	7.0
25	aromadendrene	1435	0.1
26	α-humulene	1450	0.4
27	ledene	1495	0.1
28	δ-cadinene	1518	0.1
29	caryophyllene oxide	1580	0.2
	Total identification		99.9
**Grouped components (% *v*/*v*) of *S. thymbra* essential oil**			
**Classes of EO Constituents**		**%**	
Monoterpene hydrocarbons		41.9	
Oxygenated monoterpenes		50.0	
Sesquiterpene hydrocarbons		7.7	
Oxygenated sesquiterpenes		0.2	
Aliphatic alcohols		0.1	

^a^ RI: Retention Index, calculated against C_9_–C_24_ *n*-alkanes on the HP 5MS column capillary column.

**Table 3:** Physical and chemical parameters of glycerosomes loaded with: *O. onites* essential oil (OO-GS), *S. thymbra* essential oil (ST-GS), *O. onites* plus *S. thymbra* essential oils (OOST-GS). From left: Size, polydispersity index (PdI), ζ-potential, recovery (R) and encapsulation efficiency (EE); mean ± SD (*n* = 3).

Formulation	Size (nm)	PdI	ζ-Potential (mV)	R (%)	EE (%)
OO-GS	148.40 ± 48.34	0.20 ± 0.04	−41.68 ± 5.78	87.02 ± 6.19	72.97 ± 6.46
ST-GS	105.51 ± 13.92	0.22 ± 0.05	−34.03 ± 3.57	91.38 ± 4.77	77.35 ± 7.63
OOST-GS	105.43 ± 15.19	0.17 ± 0.02	−26.50 ± 1.48	89.48 ± 5.93	73.26 ± 6.44

**Table 4:** Physical and chemical parameters of propylene glycol-nanovesicles loaded with: *O. onites* essential oil (OO-PGV), *S. thymbra* essential oil (ST-PGV), *O. onites* plus *S. thymbra* essential oils (OOST-PGV). From left: Size, polydispersity index (PdI), ζ-potential, recovery (R) and encapsulation efficiency (EE); mean ± SD (*n* = 3).

Formulation	Size (nm)	PdI	ζ-Potential (mV)	R (%)	EE (%)
OO-PGV	138.23 ± 29.17	0.17 ± 0.03	−34.93 ± 8.81	87.92 ± 8.27	83.12 ± 5.16
ST-PGV	73.95 ± 5.71	0.21 ± 0.01	−30.68 ± 6.69	84.64 ± 12.02	79.04 ± 7.34
OOST-PGV	101.09 ± 8.24	0.22 ± 0.06	−28.80 ± 1.15	85.79 ± 9.27	76.73 ± 8.27

**Table 5:** Antibacterial activity (mg EO/mL medium) of pure and formulated EOs of *O. onites* (OOEO) and *S. thymbra* (STEO).

		*B. cereus*	*S. aureus*	*L. monocytogenes*	*E. coli*	*P. aeruginosa*	*S. enterica* Serovar Typhimurium
Blank-GS	MIC	n.a.	n.a.	n.a.	n.a.	n.a.	n.a.
	MBC	n.a.	n.a.	n.a.	n.a.	n.a.	n.a.
OO-GS	MIC	0.015	0.015	0.010	0.040	0.020	0.010
	MBC	0.020	0.020	0.040	0.080	0.040	0.020
ST-GS	MIC	0.020	0.010	0.020	0.040	0.040	0.020
	MBC	0.040	0.020	0.040	0.080	0.080	0.040
OOST-GS	MIC	0.020	0.015	0.020	0.040	0.020	0.020
	MBC	0.010	0.030	0.040	0.080	0.040	0.040
Blank-PGV	MIC	n.a.	n.a.	n.a.	n.a.	n.a.	n.a.
	MBC	n.a.	n.a.	n.a.	n.a.	n.a.	n.a.
OO-PGV	MIC	0.010	0.010	0.010	0.040	0.020	0.015
	MBC	0.020	0.020	0.020	0.080	0.030	0.040
ST-PGV	MIC	0.015	0.010	0.010	0.040	0.030	0.020
	MBC	0.040	0.020	0.020	0.080	0.040	0.040
OOST-PGV	MIC	0.010	0.020	0.015	0.040	0.020	0.020
	MBC	0.020	0.040	0.020	0.080	0.040	0.040
OOEO	MIC	0.0006	0.0012	0.0020	0.0010	0.0002	0.0005
	MBC	0.0012	0.0025	0.0025	0.0020	0.0003	0.0006
STEO	MIC	0.0003	0.0012	0.0006	0.0010	0.0003	0.0010
	MBC	0.0012	0.0025	0.0012	0.0012	0.0006	0.0012
Streptomycin	MIC	0.10	0.05	0.20	0.20	0.10	0.20
	MBC	0.20	0.10	0.40	0.40	0.20	0.30

MIC: minimum inhibitory concentration; MBC: minimum bactericidal concentration; n.a.: not active at tested concentration of 8 mg formulation/mL medium, corresponding to 0.080 mg EO/mL medium; Blank-GS: blank-glycerosomes; OO-GS: glycerosomes loaded with *O. onites* essential oil; ST-GS: glycerosomes loaded with *S. thymbra* essential oil; OOST-GS: glycerosomes loaded with *O. onites* plus *S. thymbra* essential oils; Blank-PGV: blank-propylene glycol-nanovesicles; OO-PGV: propylene glycol-nanovesicles loaded with *O. onites* essential oil; ST-PGV: propylene glycol-nanovesicles loaded with *S. thymbra* essential oil; OOST-PGV: propylene glycol-nanovesicles loaded with *O. onites* plus *S. thymbra* essential oils.

**Table 6:** Antifungal activity (mg EO/mL of medium) of pure and formulated EOs of *O. onites* (OOEO) and *S. thymbra* (STEO).

		*A. fumigatus*	*A. niger*	*T. viride*	*P. verrucosum*	*C. albicans*	*C. krusei*
Blank-GS	MIC	n.a.	n.a.	n.a.	n.a.	n.a.	n.a.
	MFC	n.a.	n.a.	n.a.	n.a.	n.a.	n.a.
OO-GS	MIC	0.015	0.010	0.010	0.010	-	0.080
	MFC	0.040	0.020	0.020	0.020	-	>0.080
ST-GS	MIC	0.010	0.020	0.015	0.020	0.040	0.060
	MFC	0.040	0.040	0.030	0.040	0.080	0.080
OOST-GS	MIC	0.020	0.015	0.020	0.015	0.040	0.060
	MFC	0.040	0.030	0.040	0.030	0.080	0.080
Blank-PGV	MIC	n.a.	n.a.	n.a.	n.a.	n.a.	n.a.
	MFC	n.a.	n.a.	n.a.	n.a.	n.a.	n.a.
OO-PGV	MIC	0.010	0.020	0.020	0.020	0.060	0.080
	MFC	0.020	0.040	0.040	0.040	0.080	>0.080
ST-PGV	MIC	0.005	0.010	0.020	0.005	0.080	0.080
	MFC	0.010	0.040	0.040	0.010	>0.080	>0.080
OOST-PGV	MIC	0.020	0.010	0.020	0.015	0.080	0.060
	MFC	0.040	0.020	0.040	0.030	>0.080	0.080
OOEO	MIC	0.0002	0.0004	0.0003	0.0003	0.0010	0.0006
	MFC	0.0003	0.0006	0.0006	0.0006	0.0012	0.0012
STEO	MIC	0.0004	0.0006	0.0003	0.0003	0.0006	0.0003
	MFC	0.0006	0.0012	0.0006	0.0006	0.0012	0.0006
Ketoconazole	MIC	0.20	0.20	1.00	0.20	0.50	0.50
	MFC	0.50	0.50	1.50	0.50	1.00	1.00

MIC: minimum inhibitory concentration; MFC: minimum fungicidal concentration; n.a.: not active at tested concentration of 8 mg formulation/mL medium, corresponding to 0.080 mg EO/mL medium; Blank-GS: blank-glycerosomes; OO-GS: glycerosomes loaded with *O. onites* essential oil; ST-GS: glycerosomes loaded with *S. thymbra* essential oil; OOST-GS: glycerosomes loaded with *O. onites* plus *S. thymbra* essential oils; Blank-PGV: blank-propylene glycol-nanovesicles; OO-PGV: propylene glycol-nanovesicles loaded with *O. onites* essential oil; ST-PGV: propylene glycol-nanovesicles loaded with *S. thymbra* essential oil; OOST-PGV: propylene glycol-nanovesicles loaded with *O. onites* plus *S. thymbra* essential oils.

**Table 7:** Cytotoxic properties of pure and formulated of pure and formulated EOs of *O. onites* (OOEO) and *S. thymbra* (STEO) on human immortalized keratinocyte cell line.

Samples	IC_50_% (µg/mL) HaCaT Cell Line
Blank-GS	>500
OO-GS	311.24 ± 8.22 ^b^
ST-GS	487.34 ± 4.46 ^d^
OOST-GS	385.83 ± 1.51 ^c^
Blank-PGV	>500
OO-PGV	492.14 ± 11.74 ^d^
ST-PGV	>500
OOST-PGV	>500
OOEO	>500
STEO	>500
K_2_Cr_2_O_7_	16.29 ± 1.42 ^a^

Blank-GS: blank-glycerosomes; OO-GS: glycerosomes loaded with *O. onites* essential oil; ST-GS: glycerosomes loaded with *S. thymbra* essential oil; OOST-GS: glycerosomes loaded with *O. onites* plus *S. thymbra* essential oils; Blank-PGV: blank-propylene glycol-nanovesicles; OO-PGV: propylene glycol-nanovesicles loaded with *O. onites* essential oil; ST-PGV: propylene glycol-nanovesicles loaded with *S. thymbra* essential oil; OOST-PGV: propylene glycol-nanovesicles loaded with *O. onites* plus *S. thymbra* essential oils. Different letters mean significant difference between IC_50_ values of samples (*p* > 0.01).

**Table 8:** Preparation of OOEO-loaded vesicles.

P90G:Chol Ratio (mg/mL)	OOEO Conc (mg/mL)	Hydration Time (min)	Hydration Volume (mL)	Ultrasonic Bath	Mechanic Stirrer
15:0.5	10	30	5 (PBS)	no	yes
30:1	10	30	5 (PBS)	no	yes
30:6	10	30/60	5 (PBS)	no	yes
60:1	10	30 + 30	2.5 + 2.5 (1%G/W)	yes	yes
60:1	10	30 + 30	2.5 + 2.5 (5%G/W)	yes	yes
60:1	10	30 + 30	2.5 + 2.5 (10%G/W)	yes	yes
60:1	10	60 + 60	2.5 + 2.5 (5%G/W)	yes	yes
10:1	10	30 + 30	2.5 + 2.5 (5%G/W)	yes	yes
60:1	10	30 + 30	2.5 + 2.5 (1%PG/W)	yes	yes
60:1	10	30 + 30	2.5 + 2.5 (5%PG/W)	yes	yes

G/W = glycerol/water solution; PG/W = propylene glycol/water solution.

**Table 9:** Preparation of STEO-loaded vesicles.

P90G:Chol Ratio (mg/mL)	STEO Conc (mg/mL)	Hydration Time (min)	Hydration Volume (mL)	Ultrasonic Bath	Mechanic Stirrer
60:1	10	30 + 30	2.5 + 2.5 (5%G/W)	yes	yes
60:1	10	30 + 30	2.5 + 2.5 (1%PG/W)	yes	yes

G/W = glycerol/water solution; PG/W = propylene glycol/water solution.

**Table 10:** Preparation of OOEO plus STEO-loaded vesicles.

P90G:Chol Ratio (mg/mL)	OOEO + STEO Conc (mg/mL)	Hydration Time (min)	Hydration Volume (mL)	Ultrasonic Bath	Mechanic Stirrer
60:1	5 + 5	30 + 30	2.5 + 2.5 (5%G/W)	yes	yes
60:1	5 + 5	30 + 30	2.5 + 2.5 (1%PG/W)	yes	yes

G/W = glycerol/water solution; PG/W = propylene glycol/water solution.

## Sample Availability

Samples of the compounds are not available from the authors.
